# Steroid-Resistant Extranodal Rosai-Dorfman Disease of Cheek Mass and Ptosis Treated with Radiation Therapy

**DOI:** 10.1155/2013/428297

**Published:** 2013-05-02

**Authors:** Ahmed Marzouk Maklad, Yasser Bayoumi, Mutahir Tunio, Wafaa AlShakweer, Mashooque A. Dahar, Shomaila A. Akbar

**Affiliations:** ^1^Clinical Oncology Department, Sohag University, Sohag, Egypt; ^2^Radiation Oncology Department, NCI, Cairo University, Cairo, Egypt; ^3^Radiation Oncology Department, Comprehensive Cancer Center, King Fahad Medical City, Riyadh 59046, Saudi Arabia; ^4^Pathology Department, Comprehensive Cancer Center, King Fahad Medical City, Riyadh 59046, Saudi Arabia; ^5^Hematooncology Department, Comprehensive Cancer Center, King Fahad Medical City, Riyadh 59046, Saudi Arabia

## Abstract

*Background*. Rosai-Dorfman Disease (RDD) is rare benign hematologic disorder of histiocytes, which usually manifests as painless lymphadenopathy, fever, leukocytosis and hypergammaglobulinemia. Extranodal RDD has been reported in 43% of cases, with skin as commonly involved site followed by head and neck region. However, soft tissue cheek mass is rare presentation of extra-nodal RDD. *Case Presentation*. A 26-year-old Saudi man presented with a six-month history of right cheek swelling and left upper eyelid swelling. Physical examination revealed right cheek mass of size 3 × 3 cm and left upper eyelid mass of size 1 × 2 cm and no palpable cervical lymphadenopathy. Incisional biopsy of cheek mass showed positivity for S100 and negativity for CD1a, consistent with extra-nodal RDD. Patient did not respond to systemic steroids and was given radiation therapy to left orbit with minimal response. Then, he was started on chemotherapy Rituximab, cyclophosphamide, vincristine, and prednisolone (RCVP) 8 cycles followed by reirradiation 10 Gy in 10 fractions with 6 MeV electron with complete response. After 12 months of followup, patient had recurrence in right cheek and was started on radiotherapy to the cheek mass. *Conclusion*. Extra-nodal RDD with involvement of cheek is a rare presentation. Incorporation of S100 and CD1a is helpful in diagnoses of RDD and differentiating it from other benign histiocytosis. Treatment consists of surgery, steroids, chemotherapy, and radiation therapy.

## 1. Introduction

Rosai-Dorfman Disease (RDD) is also referred to as sinus histiocytosis with massive lymphadenopathy (SHML), which is rare, benign, and self-limited and is a non-Langerhans proliferative disorder of histiocytes [1]. It is clinically characterized by painless cervical lymphadenopathy, fever, anemia, leukocytosis, elevated erythrocyte sedimentation rate (ESR), and a polyclonal hypergammaglobulinemia [2]. It may affect any age group or any gender and without racial predilection; however, 80% of the patients are aged 20 years or younger at onset [3]. 

Extranodal RDD has been reported in 43% of cases, and the skin is the most common extranodal site [4]. However, when the disease is confined to the skin, it is classified as cutaneous Rosai-Dorfman disease (CRDD). The skin lesions have variable forms, ranging from single papules to psoriasiform, xanthomatous, acneiform, vasculitis-like, and pseudotumor-like multiple nodules and plaques; any part of the body may be affected, but more common in head and neck region [5].

Herein we presented 26-year-old Saudi man, who present with RDD of right cheek and left upper eyelid treated with steroids, systemic chemotherapy, and radiation therapy. 

## 2. Case Presentation

**Figure 1:**
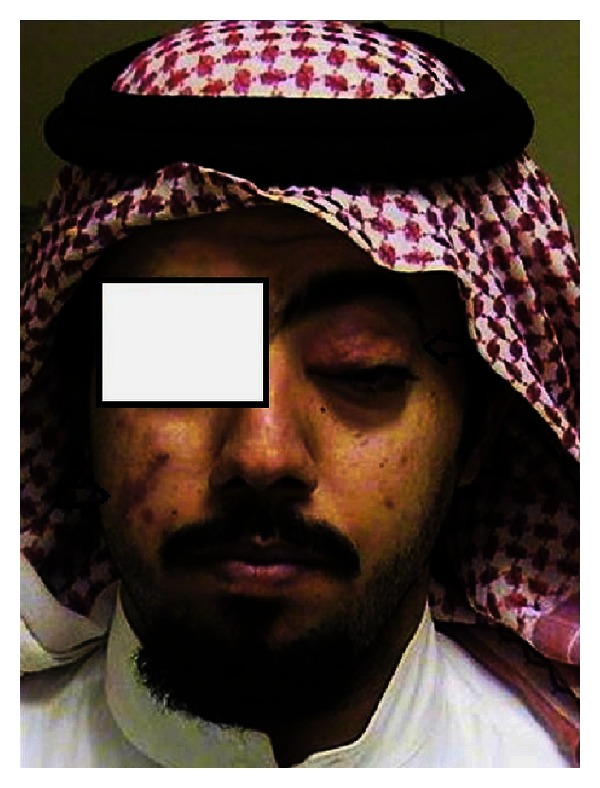
Physical examination showing right cheek erythematous lesion of size 3 × 3 cm and a similar erythematous mass over left upper medial canthus of size 1 × 2 cm with ptosis.

A 26-year-old Saudi man presented with a six-month duration of right cheek swelling and left upper eyelid swelling. Physical examination revealed right cheek erythematous mass of size 3 × 3 cm and a similar erythematous mass over left upper eyelid of size 1 × 2 cm (Figure 1); however, clinical examination revealed no lymphadenopathy or any visceromegaly. Hematology, electrolytes, serology for Lyme disease, systemic lupus erythematous, thyroid function tests, blood cultures for mycobacteria, and angiotensin-converting enzyme were all within normal limits.

**Figure 2:**
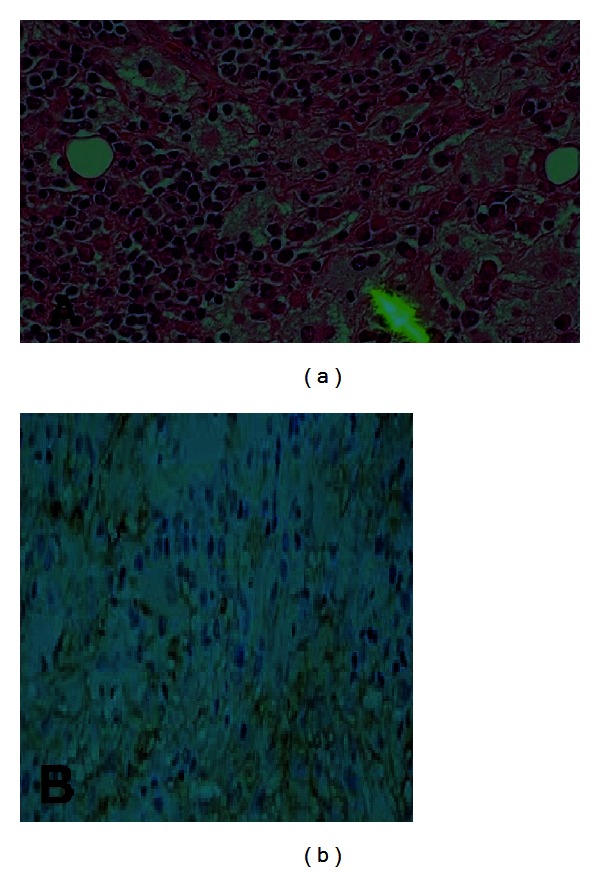
Biopsy showing (a) large histiocytes found with vesicular nuclei and emperipolesis. And (b) immunohistochemistry demonstrated the positivity for S-100, CD68, and CD45 and negativity for CD1a, consistent with the diagnosis of extra-nodal RDD.

Incisional biopsy from left eyelid mass and excisional biopsy from right cheek mass were taken, which showed a nodular inflammatory infiltrate within the dermis consisting of histiocytes with plasma cells and lymphocytes aggregates. Large histiocytes were found with vesicular nuclei and emperipolesis. Immunohistochemistry demonstrated the positivity for S-100, CD68, and CD45 and negativity for CD1a, which confirmed the diagnosis of extra-nodal RDD (Figure 2). Patient was started on oral prednisone 30 mg per day for 3 weeks and then tapered over the subsequent 2 weeks, which showed a minimal response of left eyelid mass. 

**Figure 3:**
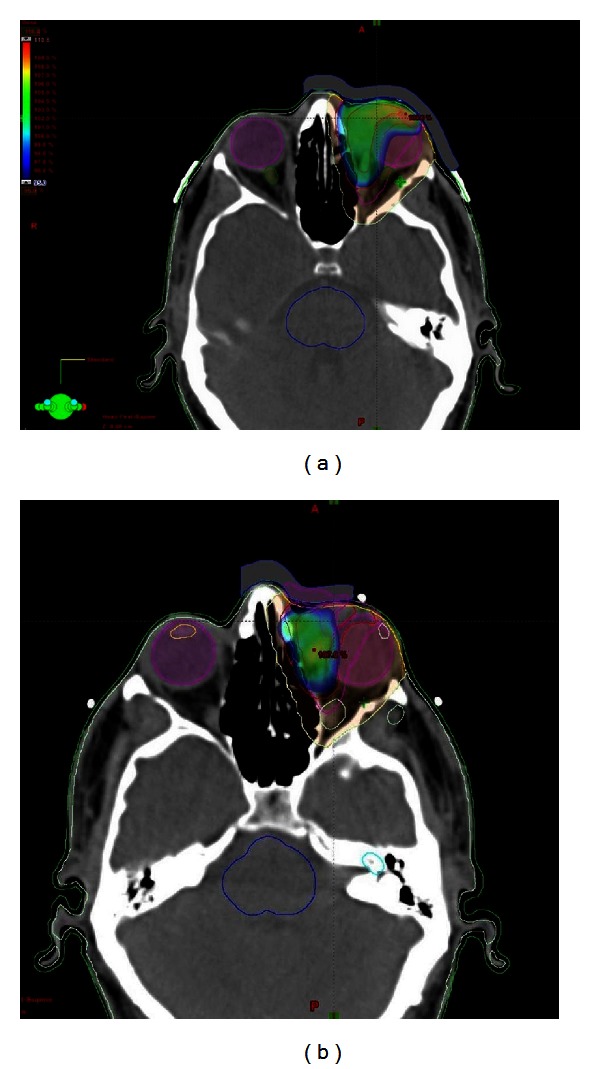
Intensity modulation radiation therapy (IMRT) to left medial canthus (a) 22.5 Gy in 15 fractions to the clinical target volume (CTV), and (b) 30 Gy in 15 fractions to the gross tumor volume (GTV) using 6 MV photons.

**Figure 4:**
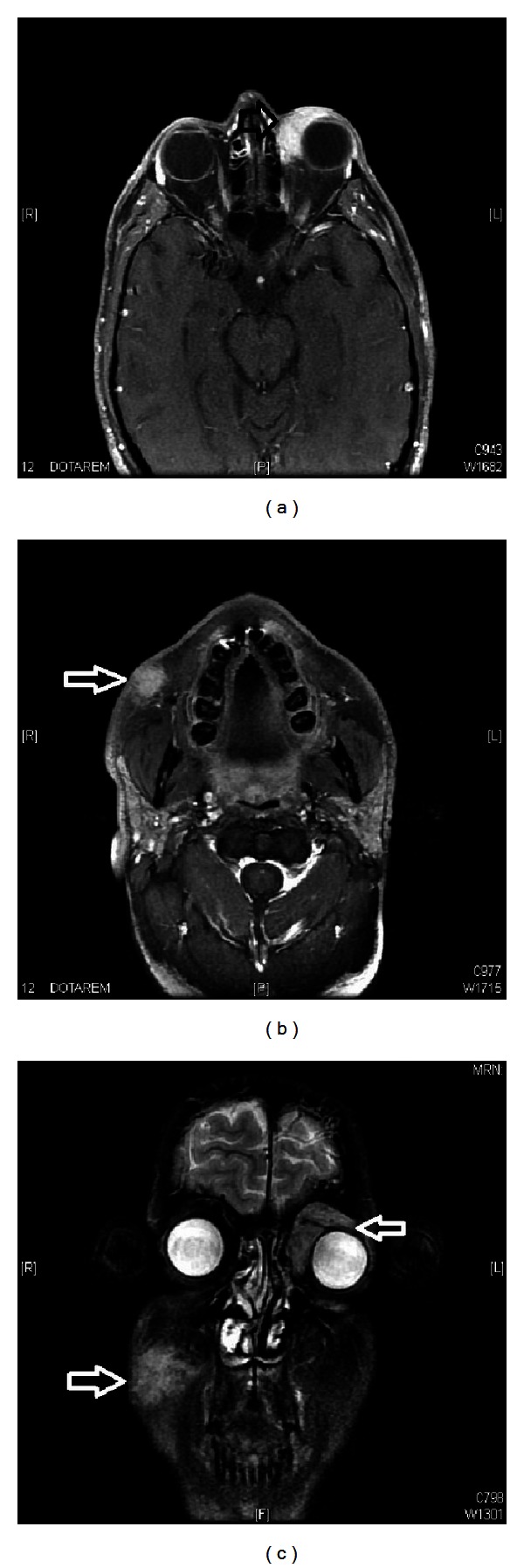
Magnetic resonance imaging (MRI) ((a) and (b)) axial and (c) coronal images of head showing recurrent mass tissue lesion involving the left eyelid, mainly superiorly and medially progressing deeply to surround the superior and medial recti muscles as well as superior oblique muscle mainly at their insertion on the ocular globe and another mass tissue lesion involving the right cheek included within the subcutaneous tissue showing intense enhancement measuring about 3 × 2 cm without obvious deep extension.

After discussing the case in multidisciplinary meeting, patient was given 30 Gy in 15 fractions radiation therapy with 6 MV photons to left eyelid mass (Figure 3). Magnetic resonance imaging (MRI) of head and neck showed more than 90% of reduction in size of eyelid lesion. After 3 months of radiation therapy, MRI of head and neck revealed the reappearance of masses in left eyelid and right cheek (Figure 4). Patient was started on Rituximab, cyclophosphamide, vincristine, and prednisolone (RCVP) based chemotherapy 8 cycles with partial response, and he was reirradiated to left eyelid mass with 6 MeV electron 10 Gy in 10 fractions with complete response. Patient remained disease-free for one year and without any late radiation induced sequelae. After one year again, there was reappearance of right cheek mass, and at the time of reporting this case report, patient was receiving radiation therapy 30 Gy in 15 fractions with 9 MeV electron beam to right cheek mass. 

## 3. Discussion

RDD is rare benign hematologic disorder of histiocytes, and its variant extra-nodal RDD is seen in 43% cases of RDD. The histological findings in extra-nodal RDD are characterized by dense infiltrate of histiocytes with scattered lymphocytes, plasma cells, and neutrophils in the dermis or subcutaneous tissue. The histiocytes are larger in size with large vesicular nuclei, small nucleoli, and abundant pale pink cytoplasm. Emperipolesis (the presence of intact lymphocytes, plasma cells, neutrophils, and red blood cells) within histiocytes is the pathognomic feature [6]. Immunohistochemistry shows the positivity for S-100 and CD68 and the negativity for CD1a [7]. These findings were also seen in our patient. 

The exact pathogenesis is not well known; however, abnormal responses of histiocytes to a viral stimuli and polyclonal expression have been postulated by different studies [8, 9]. Treatment should be based on severity of symptoms. 

No ideal treatment guidelines exist for the RDD. Many RDD lesions remain asymptomatic and heal spontaneously without any intervention [10]. Surgical excision, laser excision, steroids, liquid nitrogen, alkylating agents, thalidomide, isotretinoin and radiation therapy have been used with variable outcomes [11], as in our patient initially moderate response was achieved with steroids and 30 Gy and then complete response was made possible by alkylating agents with Rituximab and reirradiation in left eyelid and response at right cheek needs to be evaluated. 

Radiation therapy was known to have limited efficacy in most cases of RDD, although recent reports have shown benefit in a refractory extra-nodal RDD with higher radiation doses [12, 13]. However, radiation dose—response relationship and subsequent response for RDD—is not clear because of lack of reporting cases treated with radiotherapy, infrequent use of high radiation doses, and the rarity of RDD itself.

In conclusion, steroid-resistant extra-nodal RDD of cheek is rare manifestation, and multidisciplinary approach can achieve durable symptoms control. Radiation therapy can be considered as part of of RDD to enhance local control.
